# On-chip single-mode CdS nanowire laser

**DOI:** 10.1038/s41377-020-0277-0

**Published:** 2020-03-16

**Authors:** Qingyang Bao, Weijia Li, Peizhen Xu, Ming Zhang, Daoxin Dai, Pan Wang, Xin Guo, Limin Tong

**Affiliations:** 1 State Key Laboratory of Modern Optical Instrumentation, College of Optical Science and Engineering, Zhejiang University, Hangzhou, 310027 China; 2 Collaborative Innovation Center of Extreme Optics, Shanxi University, Taiyuan, 030006 China

**Keywords:** Silicon photonics, Nanowires

## Abstract

By integrating a free-standing cadmium sulfide (CdS) nanowire onto a silicon nitride (SiN) photonic chip, we demonstrate a highly compact on-chip single-mode CdS nanowire laser. The mode selection is realized using a Mach-Zehnder interferometer (MZI) structure. When the pumping intensity exceeds the lasing threshold of 4.9 kW/cm^2^, on-chip single-mode lasing at ~518.9 nm is achieved with a linewidth of 0.1 nm and a side-mode suppression ratio of up to a factor of 20 (13 dB). The output of the nanowire laser is channelled into an on-chip SiN waveguide with high efficiency (up to 58%) by evanescent coupling, and the directional coupling ratio between the two output ports can be varied from 90 to 10% by predesigning the coupling length of the SiN waveguide. Our results open new opportunities for both nanowire photonic devices and on-chip light sources and may pave the way towards a new category of hybrid nanolasers for chip-integrated applications.

In the past decade, owing to its great potential ranging from optical communications^1,2^, sensing^3^, and computing to quantum information technology^4^, on-chip nanophotonics has attracted increasing attention for the realization of integrated photonic circuits with faster operation, broader bandwidth, lower power consumption and higher compactness^5–7^. While a number of on-chip nanophotonic devices and circuits have been successfully fabricated using a complementary metal-oxide semiconductor (CMOS)-compatible technique^8^, on-chip light sources remain challenging^9,10^. On the other hand, bottom-up grown semiconductor nanowires have long been used for nanoscale waveguide lasers^11^. Benefitting from their diverse material availability and large tolerance to lattice mismatch for bandgap engineering^12–15^, nanowire lasers can now cover a broad spectral range from the ultraviolet to near-infra-red ranges^16,17^, with a number of additional advantages including compact footprints, waveguide mode quality, and excellent stability^18–20^.

In recent years, increasing attention has been paid to the integration of active nanowires with on-chip planar waveguides for on-chip light sources^21–25^. However, due to the large discrepancy in fabrication techniques, refractive index and geometric compatibility between a freestanding nanowire and an on-chip planar waveguide, a variety of issues, including a relatively low coupling efficiency, ineffective mode selection and low reproductivity, have yet to be addressed.

Relying on a highly efficient and repeatable near-field coupling approach for on-chip integration of single nanowires^26^, we demonstrate an on-chip cadmium sulfide (CdS) nanowire laser with high coupling efficiency and stability. Moreover, by forming a hybrid Mach-Zehnder interferometer (MZI) for mode selection, we operate the laser in the single-mode regime with a side-mode suppression ratio of up to a factor of 20 (13 dB). Different directional output ratios have also been achieved by predesigning the coupling length of the waveguide bends.

The structural design of the on-chip nanowire laser is schematically illustrated in Fig. 1a. A CdS nanowire is used as the gain material and is evanescently coupled to an Ω-shaped silicon nitride (SiN) waveguide at both sides to form a hybrid MZI structure. In the coupling area, SiN waveguide bends are predesigned to ensure a high coupling efficiency with excellent reproducibility^26^. The overall size of the hybrid MZI structure is kept below 100 μm. The free spectral range (FSR) of the MZI is designed to be ~1.5 nm to ensure single-mode operation. Fibre-to-chip grating couplers are designed at both ends of the SiN waveguide, which couple the laser signal from the on-chip SiN waveguide into standard optical fibres for optical characterization.

**Fig. 1:**
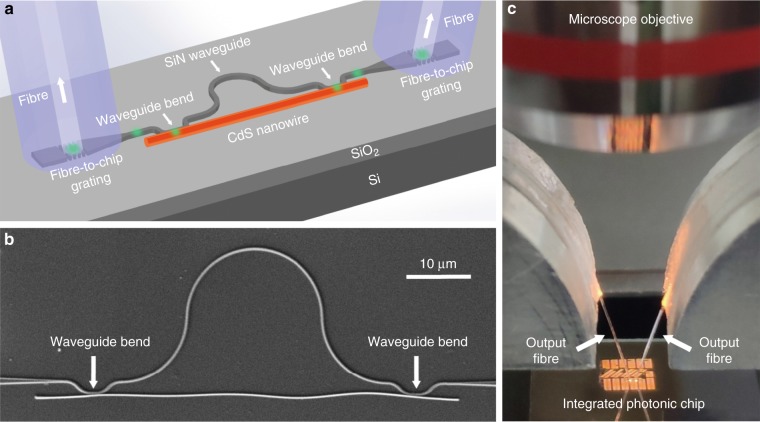
Configuration of the on-chip single-mode nanowire laser based on a hybrid MZI structure. **a** Schematic diagram and **b** SEM image of a hybrid MZI structure. The fibre-to-chip grating couplers in (**a**) are designed for coupling the laser signal into output optical fibres for optical characterization. **c** Optical image of the measurement setup under an optical microscope.

A micromanipulation process under an optical microscope (Supplementary Fig. S1) is used to integrate a CdS nanowire onto a SiN chip to form a hybrid MZI structure with excellent reproducibility. Figure 1b shows a scanning electron microscope (SEM) image of a typical hybrid MZI structure. The lengths of the CdS-nanowire arm (the partial length of the nanowire between the two coupling areas) and the SiN-waveguide arm are ~50 and 73 μm, respectively, and the coupling length of the identical SiN-waveguide bends at both sides is preset to 2.0 μm, leading to a calculated coupling efficiency of 90% for a 150-nm-diameter CdS nanowire. Figure 1c shows the measurement setup that uses output fibres to collect signals out of the chip via fibre-to-chip grating couplers.

To investigate the lasing activity of the hybrid MZI structure, we pump the CdS nanowire using 355-nm-wavelength laser pulses (duration of 3.5 ns and repetition rate of 1 kHz) above the laser threshold and measure the lasing output from one end of the nanowire. Before coupling the nanowire to the SiN waveguide, the lasing oscillation in the nanowire relies solely on the F-P cavity formed by the reflection from both ends of the nanowire (Fig. 2a), resulting in multimode lasing emission (Fig. 2b). When one side of the nanowire is coupled to the SiN waveguide (Fig. 2c), a coupling-induced spectral filtering effect^27^ may occur that produces higher loss at longer wavelengths, and additional cavities may also be introduced in the CdS nanowire for mode selection (Supplementary Fig. S2), resulting in a decrease in the mode numbers (Fig. 2d). Finally, when both sides of the nanowire are coupled to the SiN waveguide (Fig. 2e), an MZI structure is formed, which selects only one dominant lasing mode (Fig. 2f) by suppressing all other modes within the lasing spectral range (Supplementary Fig. S3), clearly showing the effectiveness of the mode selection for on-chip single-mode lasing operation.

**Fig. 2:**
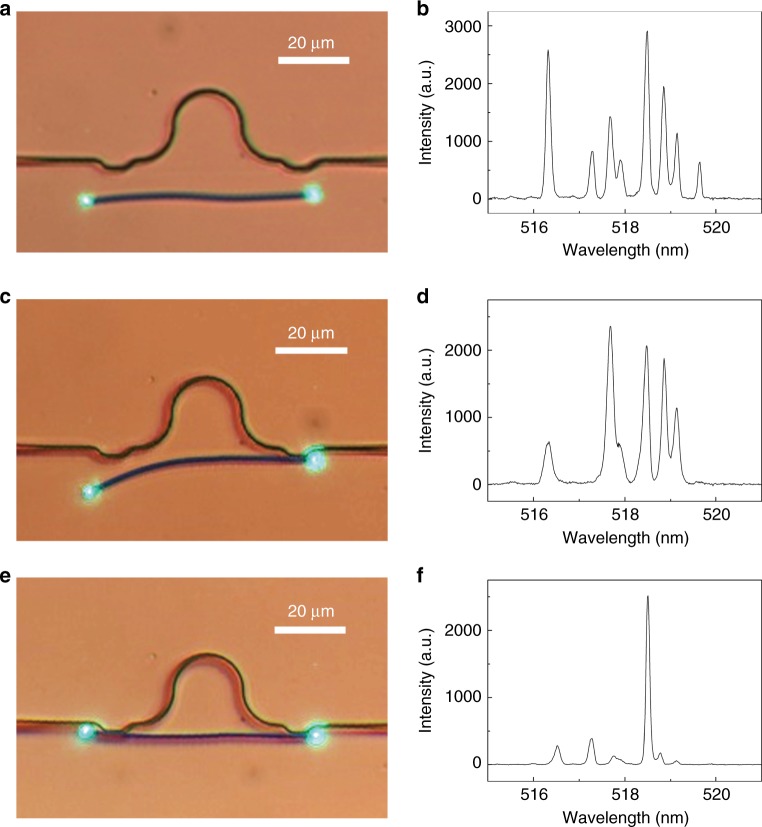
Optical characterization of a lasing CdS nanowire in different stages of the integration process. The nanowire is 300 nm in diameter and 68 μm in length and is (**a**, **b**) uncoupled, (**c**, **d**) coupled at one side, and (**e**, **f**) coupled at both sides to a SiN waveguide. **a**, **c**, **e** Optical microscope images. **b**, **d**, **f** Lasing spectra.

To characterize the on-chip nanowire laser, we measure the lasing output from the SiN waveguide via fibre-to-chip grating couplers. As shown in Fig. 3a, under optical excitation, green-colour light emission from both sides of the nanowire endfaces and the grating couplers is clearly observed. Above the lasing threshold, the measured spectra clearly show the single-mode lasing feature (Fig. 3b). The dominant lasing peak is centred at a wavelength of ~518.9 nm with a linewidth of ~0.1 nm. The side-mode suppression ratio increases with increasing pumping intensity and realizes a maximum value of approximately a factor of 20 (13 dB) at the maximum pumping intensity of 5.2 kW/cm^2^. The dependence of the lasing output on the pumping intensity (Fig. 3c) shows that the lasing threshold of the on-chip nanowire laser (∼4.9 kW/cm^2^) is slightly higher than that of the nanowire before coupling (∼4.7 kW/cm^2^), which is due to the insertion loss of the MZI for mode selection. Meanwhile, by comparing the lasing output intensities from the nanowire end and the grating area (Supplementary Fig. S4), we estimate the fractional lasing power channelled into the SiN waveguide to be ~58%, which is much higher than previous results obtained with on-chip integrated nanowire lasers or nanowire-based light emission devices^21–25^ and can be further improved by optimizing the coupling efficiency between the nanowire and the SiN waveguide.

**Fig. 3:**
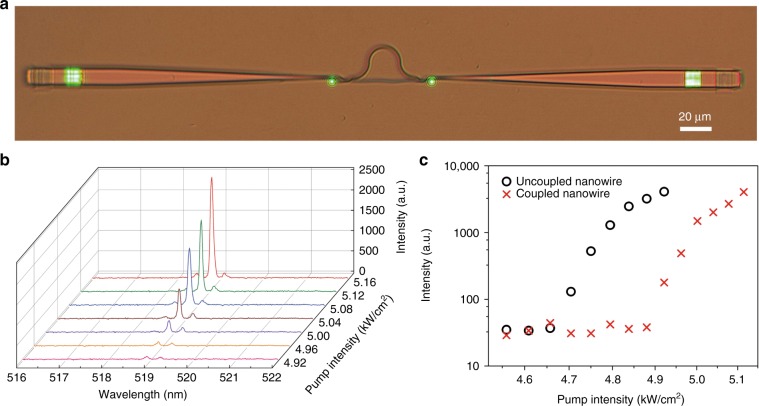
Optical characterization of an on-chip CdS nanowire laser. The nanowire is 200 nm in diameter and 65 μm in length. **a** Optical image of a hybrid MZI structure under excitation. **b** Lasing spectra obtained at different pumping intensities above the threshold. **c** Dependence of the lasing output on the pumping intensity of the excited CdS nanowire for the coupled (red) and uncoupled (black) cases.

By changing the coupling efficiency between the nanowire and the SiN waveguide and forming an asymmetric MZI lasing structure, it is also possible to adjust the ratio of the laser powers between the two directions along the SiN waveguide. To show this adjustment, we fabricate an asymmetric MZI structure with different coupling lengths for the two SiN waveguide bends that determine the coupling efficiency. Figure 4 shows a typical asymmetric MZI structure designed (Fig. 4a) and fabricated (Fig. 4b) for this purpose. The coupling lengths of the left and right SiN waveguide bends are 3.0 μm and 2.0 μm, respectively, and the CdS nanowire is 150 nm in diameter and 25 μm in length. When the asymmetric MZI structure is pumped above the threshold, the structure lases at 513.7 nm (Fig. 4c) with different output intensities for the left- and right-side gratings (Fig. 4d, e). Here, the right-side coupler achieves a coupling efficiency of ∼90% (preset value from the calculation) in channelling light from the nanowire into the SiN waveguide, and the left-side coupler is overcoupled (Supplementary Fig. S5) with a much lower efficiency (∼27%), resulting in an asymmetric output intensity between the two gratings (Fig. 4d, e). The measured ratio between the right and left grating outputs is approximately 10:1, which can be readily varied between 0.1:1 to 10:1 by adjusting the coupling lengths of the SiN waveguide bends.

**Fig. 4:**
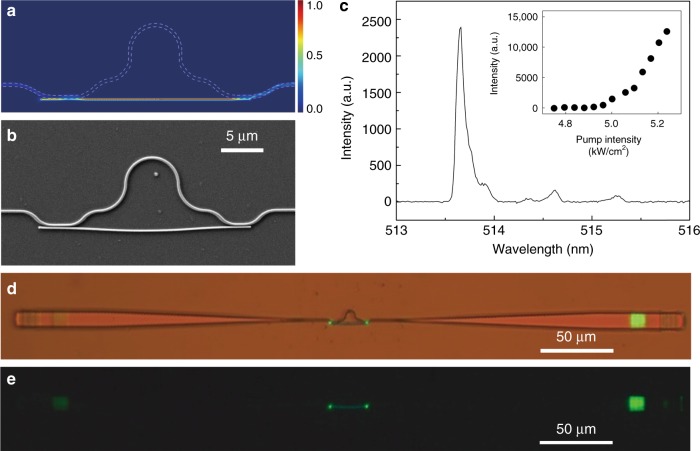
On-chip single-mode nanowire laser with an asymmetric output. **a** Numerical simulation of the coupling efficiency of an asymmetric MZI structure with different coupling lengths. **b** SEM image of an as-fabricated asymmetric MZI structure. **c** Lasing spectrum of the asymmetric-MZI-based on-chip nanowire laser. Inset: pump-intensity-dependent output intensity of the laser. **d**, **e** Optical microscope images of the laser under excitation with (upper) and without (bottom) the illumination light.

In conclusion, based on a hybrid MZI structure integrating a CdS nanowire and a SiN waveguide, we have demonstrated a new approach for on-chip lasers. Compared with previously reported chip-integrated nanowire lasers, the laser demonstrated here achieves much higher efficiency and can be operated in the single-mode regime with a small footprint and high flexibility. Benefitting from the great diversity of the available nanowire materials^17^ and high flexibility for bandgap engineering^14,15^, the on-chip integration scheme demonstrated here can be readily extended to realize on-chip nanolasers from the ultraviolet to near-infra-red ranges, which may offer new opportunities for both semiconductor nanowires and on-chip photonic devices. For example, recently, free-standing single CdS nanowires have been used for refractive index^28^ and intracellular optical sensing^29^, and the on-chip single-mode nanowire laser may thus offer an opportunity to develop on-chip physical and biochemical optical sensors with higher stability and compactness.

Experimentally, on-chip SiN waveguides are fabricated on a SiN wafer using electron beam lithography and subsequent dry etching. The as-fabricated SiN waveguides are typically 300 nm in width and 250 nm in height, with a measured waveguiding loss of less than 1 dB/cm. The CdS nanowires are synthesized using a chemical vapour deposition method^30^, which have excellent uniformities with available diameters ranging from 100 to 500 nm (Supplementary Fig. S6).

## Supplementary information

Supplementary Information for On-Chip Single-Mode CdS Nanowire Laser
