# Supplementary material for: On-chip single-mode CdS nanowire laser
Source: Light Sci Appl. 2020 Mar 16;9:42. doi: 10.1038/s41377-020-0277-0 (PMC7073330; doi:10.1038/s41377-020-0277-0)
Supplement: Supplementary file 1 — Supplementary Information for On-Chip Single-Mode CdS Nanowire Laser [file 41377_2020_277_MOESM1_ESM.docx]

## Supplementary Information for

Title:

**On-Chip Single-Mode CdS Nanowire Laser**

Qingyang Bao^1^, Weijia Li^1^, Peizhen Xu^1^, Ming Zhang^1^, Daoxin Dai^1^,Pan Wang^1^, Xin Guo^1,*^, and Limin Tong^1,2,*^

^1^State Key Laboratory of Modern Optical Instrumentation, College of Optical Science and Engineering,

Zhejiang University, Hangzhou 310027, China.

^2^Collaborative Innovation Center of Extreme Optics, Shanxi University, Taiyuan 030006, China.

*E-mail: [guoxin@zju.edu.cn](mailto:guoxin@zju.edu.cn), [phytong@zju.edu.cn](mailto:phytong@zju.edu.cn)

**1. Procedure of integrating a CdS nanowire onto a SiN chip**

CdS nanowires were manipulated by tapered fibre probes mounted on 3-dimensional moving stages under an optical microscope^1^. Firstly, using two fibre probes for micromanipulation under an optical microscope, we picked up a nanowire from an as-grown silicon wafer and transferred it onto the surface (close to the SiN waveguide) of a SiN chip (Fig. S1a). Secondly, we used the same fibre probe to push one side of the nanowire toward the SiN waveguide to make a direct contact (Fig. S1b). Then, similarly, we pushed the other side of the nanowire to contact with another SiN waveguide bend (Fig. S1c), and formed a hybrid MZI structure. Figure S1d shows the SEM image of the coupling area of a CdS nanowire and a SiN waveguide.


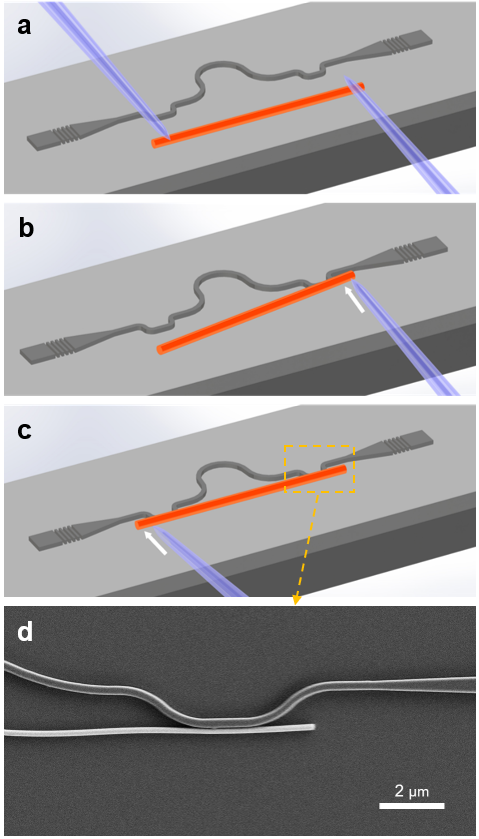


**Figure S1.** Procedure of integrating a CdS nanowire onto a SiN chip. (a) Transfer a CdS nanowire onto the surface of a SiN chip. (b) Push one side of the CdS nanowire to contact with one SiN waveguide bend. (c) Push the other side of the CdS nanowire to contact with another SiN waveguide bend. (d) SEM image of the coupling area of a CdS nanowire and a SiN waveguide.

**2. Coupling-induced spectral filtering effect**

A coupling-induced spectral filtering effect may cause higher loss at longer wavelengths, and additional cavities may also be introduced in the CdS nanowire for mode selection, resulting in a decrease in the mode numbers (Fig. S2). Cavity 1 is the original F-P cavity formed by reflection from both ends of the nanowire. When one side of the nanowire is coupled to the SiN waveguide, two new cavities are introduced by reflection from the coupling area, which are Cavity 2 and Cavity 3 shown in Fig. S2. However, this effect is not strong enough to realize the single-mode lasing operation and may result in a decrease in the mode numbers.


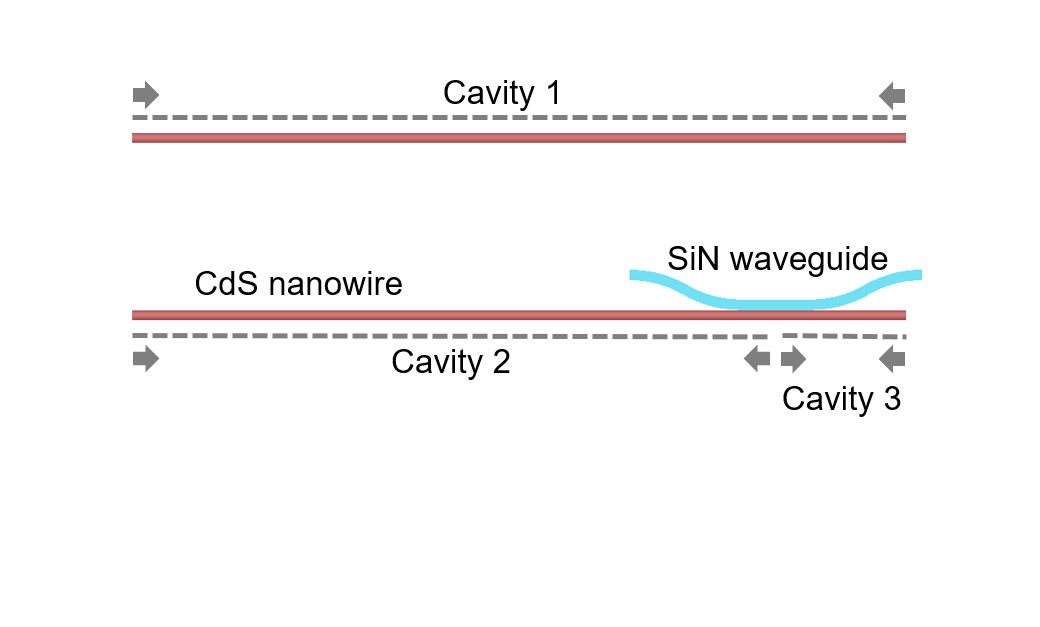


**Figure S2**. Schematic diagram of a free-standing CdS nanowire and a CdS nanowire with one side coupled to a SiN waveguide.

**3. Mode selection in a hybrid MZI structure**

Spectral response of the MZI (red dash line in Fig. S3a) is designed to offer a free spectral range (FSR) of approximately 1.5 nm. The lasing oscillation in an uncoupled CdS nanowire is multimode (black solid line in Fig. S3a). The resulting lasing oscillation after introducing the hybrid MZI structure can be estimated as the combination of the two spectra, leading to a single-mode operation (Fig. S3b).


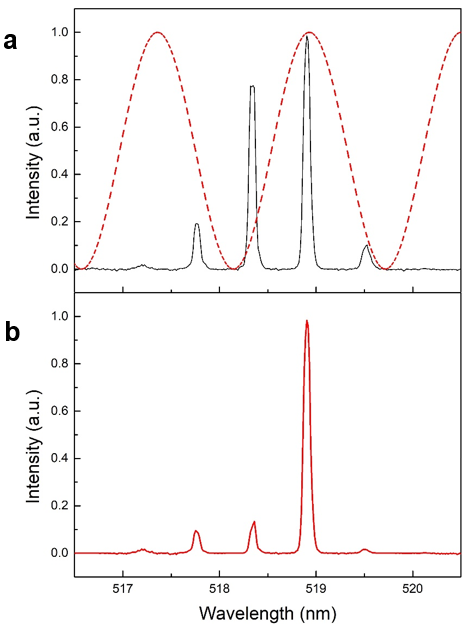


**Figure S3**. (a) Spectral response of a hybrid MZI (red dash line) and lasing spectrum of an uncoupled CdS nanowire (black solid line). (b) Resulting lasing oscillation estimated as the combination of the two spectra.

**4.** **Estimation of coupling efficiency of channelling light from a CdS nanowire into a SiN waveguide**

We take the gray values of the microscope images and integrate the gray values of the light spots at the endfaces of the nanowire and the gratings (Fig. S4a, Fig. S4b). To eliminate the background, we make a difference between the bright area and the nearby dark area and use the difference value as the intensity of the laser output. By directly comparing the lasing output intensities from the CdS nanowire endface and the grating coupler, we obtain an intensity ratio between the SiN waveguide and the CdS nanowire to be approximately 10:1.

To calibrating the collection efficiency, we use Lumerical FDTD to simulate the collecting efficiencies of the light radiation of the SiN waveguide grating coupler and the CdS nanowire endface into the far field^2^. Figure S4c is the numerical simulation of radiation output power intensity distribution at 520-nm wavelength of a grating coupler. Figure S4d is the numerical simulation of endface output power intensity distribution at 520-nm wavelength of a 200-nm-diameter CdS nanowire. Here we use a microscope objective with numerical aperture (NA) of 0.35 (the same as we used in the experiment), within which we collect radiation with efficiencies of 28.5% from the grating coupler (Fig. S4e) and 3.9% from the nanowire endface (Fig. S4f), respectively. After calibrating the collecting efficiency, we obtain a coupling efficiency of approximately 58% for channelling lasing signal from the CdS nanowire into the SiN waveguide.


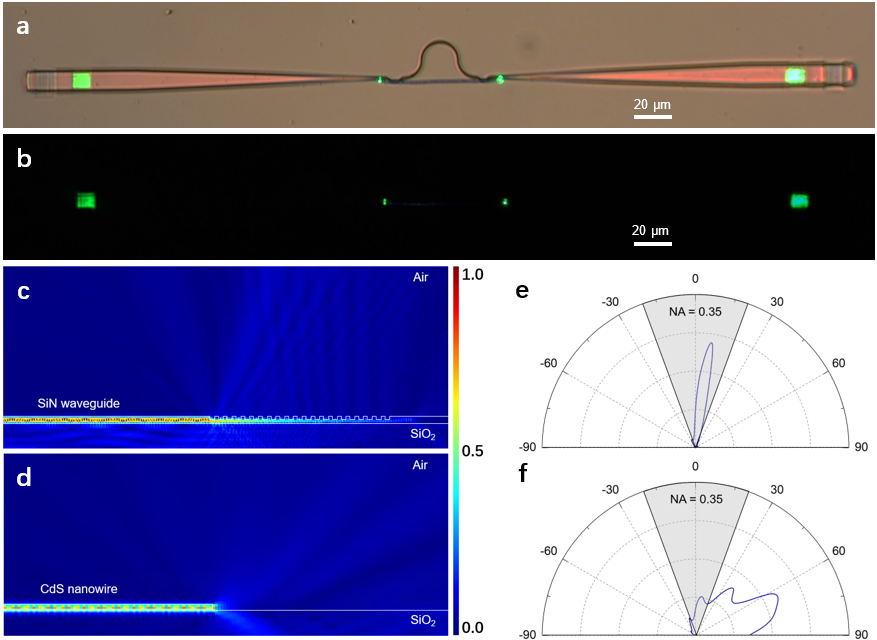


**Figure S4.** Optical images of a hybrid MZI structure with lumination light (a) on and (b) off. (c) Numerical simulation of radiation output power intensity distribution at 520-nm wavelength of a grating coupler. (d) Numerical simulation of endface output power intensity distribution at 520-nm wavelength of a 200-nm-diameter CdS nanowire. (e) Angular distribution of scatterd light extracted from (c). (f) Angular distribution of scatterd light extracted from (d). Collecting angles with NA of 0.35 are indicated in (e) and (f).

**5. Simulation of the coupling efficiency between a waveguide bend and a CdS nanowire**

We use Lumerical FDTD to simulate the near-field optical coupling between a CdS nanowire and a SiN waveguide bend. Power intensity distributions on horizontal xz planes across the central axes of the SiN waveguide and the nanowire are depicted in Fig. S5. When the length of the waveguide bend is 2.0 μm for optimized coupling, the calculated coupling efficiency is 90% (Fig. S5a). When the length of the waveguide bend is 3.0 μm for over-coupling, the calculated coupling efficiency decreases to 27% (Fig. S5b).


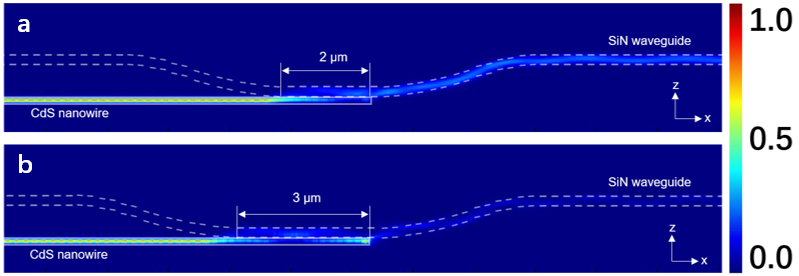


**Figure S5.** Numerical simulation of power intensity distributions on horizontal xz planes across the central axes of the SiN waveguide and the CdS nanowire. (a) Waveguide bend with a length of 2.0 μm for optimized coupling. (b) Waveguide bend with a length of 3.0 μm for over-coupling.

**6. Fabrication of SiN waveguides and synthesis of CdS nanowires**

The on-chip SiN waveguides and structures were fabricated on a SiN wafer with a 250-nm-thick top SiN layer and a 3-μm-thick buried oxide layer. After spin-coating photoresist on the wafer, we used an electron beam lithography process to define SiN waveguide patterns to the photoresist, followed by an inductively coupled plasmon dry-etching process to fully etch the top SiN layer down to the buried oxide layer with the photoresist mask. To facilitate direct contact between a CdS nanowire and a SiN waveguide for better optical coupling, the SiN waveguides are exposed in air (i.e., air-cladded). Figure S6a and Figure S6b show the optical and SEM images of typical as-fabricated SiN waveguides. Figure S6c shows the close-up SEM image of a typical as-fabricated grating coupler. The etch depth of the grating coupler is 150 nm and the pitch is 340 nm with a duty cycle of 0.5.

The CdS nanowires were grown using a chemical vapor deposition method^3,4^. Here the growth was conducted by evaporating CdS powder at 800 °C in a quartz tube furnace, with a high-purity nitrogen flow as the carrier gas. Small pieces of silicon wafers coated with Au film were placed downstream of the CdS powder to collect the grown nanowires. After 1.5-h growth, the tube was cooled down to room temperature and the silicon wafers with as-grown nanowires were taken out for experiments. Figure S6d and Figure S6e show the SEM images of the CdS nanowire.

**
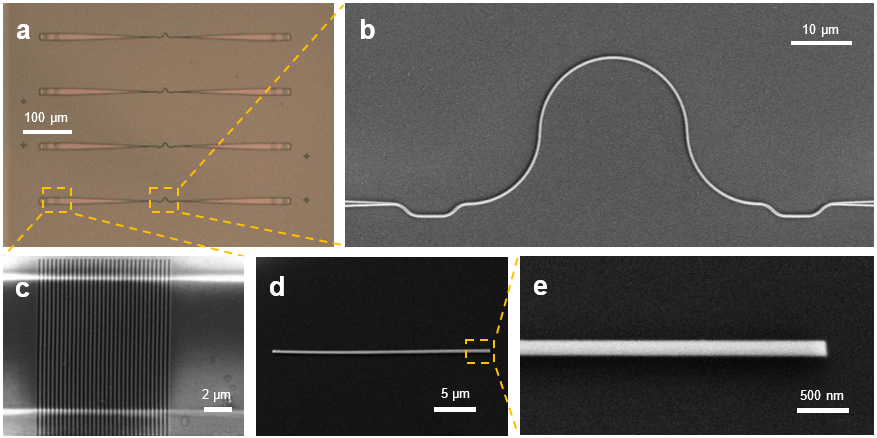
**

**Figure S6.** (a) Optical microscope image of SiN waveguide arrays. (b) Close-up SEM image of a SiN waveguide shown in (a). (c) Close-up SEM image of a grating coupler shown in (a). (d) SEM image of an as-grown CdS nanowire. (e) Close-up SEM image of one endface of the nanowire shown in (d).

Supplementary information accompanies the manuscript on the Light: Science & Applications website (<http://www.nature.com/lsa/>)

**References**

1. Guo, X. *et al*. Photonic nanowires: from subwavelength waveguides to optical sensors. *Accounts of Chemical Research* **47**, 656-666 (2014).
2. Oulton, R. F. *et al.* Plasmon lasers at deep subwavelength scale. *Nature* **461**, 629-632 (2009).
3. Xia, Y. N. *et al.* One dimensional nanostructures: synthesis, characterization, and applications. *Advanced Materials* **15**, 353-389 (2003).
4. Fan, H. J. *et al.* Semiconductor nanowires: from self-organization to patterned growth. *Small* **2**, 700 (2006).
